# Toward universal screening for disease-causing alleles: Mendelian susceptibility to mycobacterial disease as a model

**DOI:** 10.70962/jhi.20250094

**Published:** 2025-09-29

**Authors:** Farida Almarzooqi, Ahmed Aziz Bousfiha

**Affiliations:** 1 Department of Internal Medicine, College of Medicine and Health Sciences, United Arab Emirates University, Alain, United Arab Emirates; 2 Laboratoire d’Immunologie Clinique, Infectiologie et Autoimmunité, LICIA, Faculty of Medicine and Pharmacy, King Hassan II University, Casablanca, Morocco; 3 Department of Pediatric Infectious Diseases and Clinical Immunology, A. Harouchi Hospital, Ibn Rochd Medical School, Casablanca, Morocco

## Abstract

Advances in genomic technologies, including whole exome and genome sequencing, have transformed diagnosis of monogenic disorders such as inborn errors of immunity (IEIs). In high-consanguinity populations like the United Arab Emirates (UAE), where autosomal recessive disorders are prevalent, early genomic screening shifts care from reactive diagnosis to personalized care. UAE national programs remain limited to premarital or neonatal panels, missing disorders with variable onset or incomplete penetrance. We advocate universal genomic screening to integrate disease-causing alleles into clinical care. As proof of concept, we highlight Mendelian susceptibility to mycobacterial disease (MSMD), an IEI defined by impaired interferon-gamma signaling and severe complications following *Bacillus Calmette–Guérin* (BCG) vaccination and *Mycobacterium tuberculosis* disease. We propose a tiered approach using MSMD-related genes within exome or genome platforms, enabling scalable, cost-effective implementation and periodic reanalysis as evidence evolves. In the UAE, high consanguinity, genomic infrastructure, and regulatory frameworks position MSMD as an entry point for population genomic screening, advancing precision medicine and prevention.

## Teaser

This Perspective proposes universal genomic screening as a life-course strategy, using Mendelian susceptibility to mycobacterial disease as a model to demonstrate feasibility in high-consanguinity populations, where early genetic detection can enhance diagnosis, improve vaccine safety, and advance precision medicine.

## Introduction

Advances in genetic testing, particularly whole exome and whole genome sequencing (WES/WGS), have revolutionized the diagnosis of both rare and common genetic conditions in the population. These technologies enable the comprehensive detection of variants, thereby enhancing diagnostic precision and supporting early, targeted interventions (1, 2). Among their most transformative applications is the identification of inborn errors of immunity (IEIs), a rapidly expanding group of over 500 monogenic immune disorders (3). These advances have deepened our understanding of IEIs, which manifest through a wide spectrum of clinical features, including recurrent infections, immune dysregulation, autoimmunity, autoinflammation, and malignancy (4, 5, 6). However, early recognition remains challenging in high-consanguinity populations due to increased homozygosity and nonspecific IEI presentations.

In the Arab region, consanguinity rates can reach up to 50% in certain subpopulations, such as in Saudi Arabia and the United Arab Emirates (UAE) (7). This cultural practice significantly contributes to the high prevalence of autosomal recessive disorders, including thalassemia, congenital anomalies, cardiovascular defects, and primary immunodeficiencies (8). The Emirati population (UAE nationals) represents about 11.5% of the country’s total population and is marked by genetic homogeneity due to high rates of intermarriage (8, 9, 10). While not culturally mandated, this pattern increases the population’s susceptibility to autosomal recessive monogenic disorders.

To address the burden of inherited disorders, the UAE has implemented national genomic initiatives, including premarital and newborn screening programs, to enable early detection and prevention (11, 12, 13). However, these current models remain targeted and fragmented. Premarital screening focuses on identifying carrier couples for selective high-prevalence disorders, primarily addressing reproductive risk. Newborn screening, while essential, is limited to a fixed panel of treatable conditions within the neonatal period. These programs operate in isolation and lack predictive capacity for variable, or late-onset genetic disorders. They often miss subtle or atypical cases by overlooking incomplete penetrance, heterozygous effects (in autosomal recessive [AR] disorders), and gene–environment interactions, resulting in delayed diagnoses and missed opportunities for early and personalized intervention (14, 15).

This gap calls for a more integrated, life-course genomic strategy. A universal genetic screening framework could bridge existing gaps, incorporate predictive analytics, and enable personalized health planning from birth through adulthood. This commentary advocates for universal genomic screening as a foundational strategy to integrate known disease-causing alleles into routine clinical care. As a proof of concept, we highlight Mendelian susceptibility to mycobacterial disease (MSMD), a form of IEI, as a model to demonstrate the feasibility and impact of genomic screening in high-consanguinity populations.

## Model-based perspective: MSMD as a model

MSMD is a rare primary immunodeficiency caused by monogenic IEIs that impair interferon-gamma (IFN-γ)–mediated immune responses, increasing susceptibility to weakly virulent mycobacteria, such as *Mycobacterium bovis*-*Bacillus Calmette–Guérin* (*BCG*), *Mycobacterium tuberculosis*, and environmental mycobacteria (16, 17). Mutations in genes, including *IFNG*, *IFNGR1*, *IFNGR2*, *IL12RB1*, tyrosine kinase 2 (*TYK2*), signal transducer and activator of transcription 1 (*STAT1*), and *CYBB*, lead to diverse clinical phenotypes, ranging from localized BCG-itis to disseminated mycobacterial disease and tuberculosis (TB) (16). In a systematic review, *BCG*-related complications occurred in over 84% of vaccinated MSMD patients, often presenting with lymphadenopathy, osteomyelitis, fever, and systemic symptoms. Beyond mycobacterial infections, MSMD patients may also develop non-typhoidal salmonellosis and chronic mucocutaneous candidiasis (16, 17, 18). While MSMD primarily predisposes individuals to less virulent mycobacteria, certain variants (e.g., *IL12RB1* and *TYK2*) have also been linked to TB as the sole phenotype, supporting a genetic basis for TB susceptibility (18).

## UAE as case

Given the high consanguinity rate in the UAE, the population is at increased risk for autosomal recessive IEIs, including MSMD (particularly forms caused by variants in *IL12RB1*, *IFNGR1*, *STAT1*, *IL12B*, *IFNGR2*, *TYK2*, RAR-related orphan receptor C (*RORC*), *IL23R*, *SPPL2A*, *ISG15*, *ZNFX1*, *IRF8*, *IL12RB2*, *USP18*, *IFNG*, *JAK1*, *TBX21*, and *IRF1*) (16). In countries like the UAE, where *BCG* vaccination is mandatory at birth, identifying these variants is critical for early diagnosis, tailored infection prevention, and TB risk stratification, as well as for guiding treatment decisions such as antimycobacterial therapy, IFN-γ administration, and hematopoietic stem cell transplantation in severe case (16, 19).

At present, there are no published epidemiological data estimating the frequency of MSMD or the prevalence of deleterious MSMD alleles in the UAE. Furthermore, a public genomic database reporting allele frequency for MSMD-related genes is lacking. Despite this, the UAE’s high consanguinity and genetic overlap with neighboring Gulf and Arab countries suggest a similar burden of autosomal recessive MSMD (7). A multicenter retrospective study from the Gulf region, including Tawam Hospital in the UAE, examined *BCG* vaccine-related complications in patients with combined immunodeficiencies (CIDs) (20). Despite established contraindications, many were still vaccinated. Similar complications have also been reported in Emirati newborn case reports, underscoring ongoing risks (21, 22, 23). Together, these observations reinforce the need for IEI newborn screening, postponing *BCG* vaccination beyond 6 months, and adopting safer BCG strains, particularly in countries with a high incidence of CID such as the UAE (20).

Regional data also strongly support the relevance of MSMD in high-consanguinity settings. In Saudi Arabia, which shares similar population genetics with the UAE, a recent review reported 54 confirmed MSMD cases, ranking it among the top five countries globally. This underlines the combined impact of founder variants and universal neonatal *BCG* vaccination in unmasking MSMD. In another study, 66% of Saudi infants with disseminated *BCG* disease were found to have IL-12 deficiency, and 41% were from consanguineous families (24). A multinational review of IL-12p40 deficiency, including Saudi patients, showed that 97.5% of vaccinated individuals developed *BCG*-related disease and had non-typhoidal salmonella infections being more common than mycobacterial relapses (25). In response, Saudi Arabia’s national policy to delay *BCG* vaccination to 6 months significantly reduced complications in severe CID patients (26). Similar patterns have been observed elsewhere in the region; for example, in Morocco, 55% of MSMD patients experienced *BCG*-related complications, commonly associated with variants in *IL12RB1*, *STAT1*, and *TYK2* (24). In Oman, disseminated *BCG* disease occurred in 4.9% of vaccinated children, with 72.7% having underlying primary immunodeficiencies (27). These findings underscore the regional impact of MSMD and support the importance of implementing early genetic screening in populations with high consanguinity and mandatory *BCG* vaccination (28).

The UAE, while part of this high-risk regional context, presents a unique case: it maintains one of the lowest TB incidence rates in the World Health Organization Eastern Mediterranean Region (<1 case per 100,000 population as of 2021) (29). However, despite its low TB incidence, the UAE faces ongoing risk due to the high inflow of individuals from TB-endemic countries, particularly labor migrants (30, 31, 32). Thus, UAE offers a valuable platform to study the association between mycobacterial diseases and MSMD. Given the high consanguinity rate and associated risk of autosomal recessive immune disorders, investigating MSMD in this genetically vulnerable, low-TB-incidence setting provides a compelling basis for our hypothesis. As a proof of concept, MSMD highlights how early detection of mycobacterial-related genetic susceptibility could inform targeted screening and preventive health strategies. Collectively, regional data suggest MSMD is likely underrecognized in the UAE and underpin the clinical relevance of AR-MSMD genes in this population. While we acknowledge the rarity of MSMD and the current absence of national incidence data, we highlight the need for population-based studies to validate this approach and inform future health policy.

## Tiered genomic approach

To implement this model, we propose a tiered genomic screening framework beginning with a soft gene-enriched panel (a flexible, data-driven gene list used to filter or prioritize candidate disease genes from WES/WGS) targeting MSMD-related genes (e.g., *IFNGR1*, *IFNGR2*, *IL12RB1*, *TYK2*, *STAT1*, and *RORC*) (33). This panel draws from global databases, such as the Online Mendelian Inheritance in Man (OMIM) and International Union of Immunological Societies, and can be further refined with local genomic and clinical data. Integrated within WES/WGS platforms, this bioinformatic filter allows for cost-effective, focused first-tier analysis while retaining flexibility for reanalysis as gene–disease associations evolve (33, 34). This soft panel approach avoids the limitations of custom re-sequencing chips, which is physical, lab-based assays optimized for predefined gene sets, and instead supports scalable, phenotype-driven variant interpretation (1, 35). If no actionable variant is detected through the soft panel, the broader WES/WGS data will be reanalyzed to explore potential novel or atypical variants associated with immune risk, allowing efficient prioritization while retaining the capacity to investigate rare or unexpected findings in greater depth.

Variants of uncertain significance (VUS) can be identified during the initial tier of analysis. Uncertainty persists even within restricted sets of high-priority genes, such as those implicated in MSMD, due to incomplete functional annotation, evolving genotype–phenotype relationships, and limited representation of diverse populations in genomic reference databases (36, 37, 38). Large-scale studies demonstrate that VUS are reported in ∼30% of multigene panel tests, with frequencies reaching up to 50% in MSMD-related cohorts (36, 39). Comparable findings have been observed in exome sequencing studies of IEIs, where systematic reanalysis has been shown to improve diagnostic yield and classification accuracy (40).

In our model, VUS will be securely archived within institutional databases and managed through a structured ethical framework that supports traceable reanalysis in line with evolving gene–disease evidence (41, 42). Reanalysis will be operationalized in the following steps: (1) VUS are flagged and archived during the initial analysis; (2) scheduled or trigger-based re-evaluations will apply American College of Medical Genetics and Genomics/Association for Molecular Pathology (ACMG/AMP) and Clinical Genome Resource (ClinGen) criteria using updated population data, functional evidence, and clinical correlations; and (3) candidate reclassifications will undergo multidisciplinary review before issuing amended reports (43, 44).

To support the interpretation and prioritization of candidate variants, our approach will employ a suite of validated in silico prediction tools, including combined annotation-dependent depletion, rare exome variant ensemble learner (REVEL), polymorphism phenotyping v2, sorting intolerant from tolerant, mutationtaster, and mutation significance cutoffs (37, 38, 40). These algorithms incorporate diverse predictive features, such as evolutionary conservation, predicted effects on protein structure and function, and allele frequency data, to estimate pathogenicity. Their application is especially valuable during the early phases of variant triage when functional validation is not yet feasible. Consensus across multiple predictors improves interpretive confidence, with benchmarking studies showing that meta-predictors, such as REVEL and Bayesian deleteriousness, generally outperform individual tools, with gene-specific thresholds offering further refinement (45). In alignment with ACMG/AMP and ClinGen guidelines, computational predictions are incorporated as supportive evidence within standardized variant classification frameworks, but their interpretation remains provisional unless validated by functional data (46).

Guided by the same ACMG/AMP and ClinGen recommendations, VUS identified through this process will not be used to guide clinical decision making at the time of detection. Instead, genetic counseling will emphasize their nonactionable and nondiagnostic status while clearly communicating the potential for future reclassification (37, 44, 47, 48, 49). Embedding this structured approach into the tiered genomic screening framework preserves analytical integrity, enhances transparency, and supports long-term data stewardship.

Crucially, our model underscores rare, high-impact variants in established MSMD-related genes to predict risk for mycobacterial disease. These predictions rely on bioinformatic platforms and curated databases (e.g., OMIM and ClinVar) to support clinically meaningful genotype–phenotype correlations. Importantly, we will exclude low-effect common variants (e.g., odds ratio <10), which have limited predictive utility in individual risk assessment, as emphasized in previous critiques of polygenic risk score models for complex trait (50, 51). In our model, such low-effect variants are excluded, and our focus remains on high-impact variants in established MSMD-related genes, where predictive value is clinically meaningful and relevant to public health decisions.

## Clinical integration

Sequencing data will be securely stored within national health information systems. Dedicated bioinformatic pipelines will annotate variants based on zygosity, inheritance pattern, and clinical significance, using standardized identifiers such as OMIM and ClinVar. Where appropriate, individualized genomic risk profiles can be stratified by clinical urgency and directed to the most relevant healthcare providers. Referrals may include general practitioners for primary care coordination, infectious disease specialists for managing pathogen-related susceptibilities such as mycobacterial infections, and community medicine physicians for broader public health follow-up. Such a referral model ensures that clinically actionable findings are embedded into care pathways and translated into timely, personalized interventions. To support successful implementation, structured training programs should be provided for healthcare professionals, focusing on variant interpretation, Mendelian inheritance, and effective communication of results. Complex or uncertain cases should be referred to clinical geneticists to ensure accurate diagnosis and appropriate counseling.

## Cost-effectiveness

To support the rationale, we have incorporated regional estimates and published case series (e.g., from Saudi Arabia, Oman, and Morocco), which suggest a likely underrecognized burden of MSMD in the UAE due to similar genetic and clinical risk factors, particularly high consanguinity (20, 21, 22, 23, 24, 25, 26, 27, 28). We underline that pilot studies, registry development, and cost-effectiveness modeling will be essential next steps.

Although genomic screening may involve a substantial upfront investment, it is increasingly feasible and potentially cost-effective in the UAE due to several contextual advantages. These include a relatively modest annual birth cohort (∼90,000 births), a well-established national newborn screening infrastructure, and significant government investment in genomic medicine through initiatives such as the Emirati Genome Program (52, 53). Regional data (from Oman) estimate the incidence of *BCG*-related complications at 9.2 per 100,000 vaccinated neonates, which translates to ∼8–9 cases annually (54, 55). Of these, 15–45% may involve underlying IEIs such as MSMD (54, 55). The economic burden of treating such complications is significant. A recent cost analysis from a tertiary hospital in Abu Dhabi, UAE, found that inpatient management of suspected TB cases ranged from $9,075 to $11,140 per patient (56). In contrast, managing disseminated *BCG *infection may exceed $100,000 per case when accounting for prolonged hospitalization, IFN-γ therapy (∼$21,840 per patient annually), and potential hematopoietic stem cell transplantation (in IEI patients) (56, 57).

WES currently costs approximately AED 7,350 (∼USD 2,000) per individual in the UAE through accredited providers (58). Meanwhile, the Emirati Genome Program, launched in Abu Dhabi, UAE, and providing free genomic testing for Emirati nationals, is now expanding nationwide. As the program expands, it is expected to significantly reduce the cost of population-level screening, particularly for targeted panels relevant to MSMD-associated genes (53). In this context, identifying even 3–5 high-risk infants annually through genomic screening could result in significant cost savings and reduce preventable morbidity (52, 54, 55). Thus, early identification of at-risk individuals through genomic screening enhances clinical outcomes, reduces diagnostic delays, and guides timely interventions such as deferring *BCG* vaccination in vulnerable infants. It also supports TB risk stratification and aligns with broader public health goals by preventing avoidable complications and minimizing misdiagnosis. Even averting a small number of severe cases annually could offset substantial healthcare costs and demonstrate the value of integrating genomic tools into national health strategies.

MSMD exemplifies how population-specific genomic screening can guide evidence-based decisions, not only for *BCG* vaccination and TB prevention, but also for family counseling and long-term care planning. In high-consanguinity populations, where autosomal recessive IEIs are more prevalent, early detection reduces reliance on intensive care and enables targeted, timely intervention (59).

Building on existing national genomic initiatives, we propose expanding this model into a broader population-level framework for managing inherited immunological risk. Integrating genomic screening into routine care shifts clinical practice from reactive treatment to preemptive precision-based prevention. This approach benefits not only MSMD patients but also individuals with other IEIs by improving outcomes, accelerating diagnosis, avoiding unnecessary testing, and lowering long-term healthcare expenditures (60). Genomic screening, as a one-time early-life intervention, supports sustainable, population-scale strategies for lifelong health planning.

## Life-course perspective

While many MSMD cases are identified in infancy, particularly following *BCG* vaccination, increasing recognition of adult-onset MSMD highlights the need for a life-course screening model. Adult-onset cases, often triggered by exposure to *M. tuberculosis* or environmental mycobacteria, are frequently missed due to nonspecific symptoms and normal immunologic workups (61). Reported genetic etiologies in adult-onset MSMD include *IL12Rβ1*, *IFNγR1*, *TYK2*, *NEMO*, and *STAT1* deficiencies. Incomplete penetrance, such as only 50–70% of *IL12Rβ1*-deficient individuals being symptomatic by age 40, highlights the need for sustained genomic risk awareness across the life course (62). MSMD-based genomic screening frameworks, therefore, offer a practical model for detecting genetically predisposed individuals across the lifespan, not just in infancy but also in adulthood upon TB exposure.

## Ethical and legal considerations

While genomic screening holds significant promise for advancing personalized medicine, improving early diagnosis, and informing public health policy, it also presents complex ethical, legal, and social challenges that demand careful consideration. Key concerns include data privacy, long-term storage, and potential reanalysis of genomic data, all of which necessitate robust legal safeguards and responsible data stewardship. Informed consent is central, yet the complexity of genomic information often makes it difficult for parents to fully understand potential outcomes, including incidental or uncertain findings. This raises further debate over the appropriate scope of results, whether to return only actionable, childhood-onset conditions or to include adult-onset or nonactionable variants, potentially impacting the child’s future autonomy. Additionally, there is a risk of stigmatization and genetic discrimination if protections are not clearly defined. Equitable access must be prioritized to prevent genomic screening from exacerbating existing health disparities. Ultimately, successful implementation requires comprehensive regulatory frameworks that balance public health benefits with individual rights, ensuring that screening is delivered ethically, responsibly, and fairly. Current international guidelines recommend that only clinically actionable results be returned, and they highlight the need for transparent governance over long-term data storage, sharing, and secondary use (63, 64, 65).

In the UAE, existing legal infrastructure provides a strong foundation for ethical implementation. Medical Liability Law (Federal Law No. 4 of 2016) and the Health Data Law of 2021 regulate the use of patient data, ensuring privacy, informed consent, and controlled access (66, 67). Further strengthening this framework, Federal Law of 2023 specifically governs the use of human genomic data, mandating the creation of a National Genomic Database and Emirati Genome Reference, while prohibiting unethical uses such as cloning or unauthorized gene modification (68). This law enforcers strict safeguards for data protection, aligns with international bioethical principles, and reinforces the UAE’s commitment to responsible innovation in genomic medicine.

## UAE infrastructure readiness

In parallel with strong legal safeguards, the UAE’s existing newborn screening infrastructure provides a practical foundation for implementation. The UAE’s National Newborn Screening Program, launched in 1995, already offers a successful model for early detection. It currently screens for over 40 genetic and metabolic conditions, including phenylketonuria, congenital hypothyroidism, sickle cell disease, and G6PD deficiency, along with universal hearing and congenital heart screening. Over 1.3 million infants have been screened, leading to the early diagnosis of more than 2,700 serious cases. This robust infrastructure, combined with high parental acceptance, provides an ideal platform for the ethical and efficient expansion to genomic screening (69). Practical experience from 2011 to 2014 with inborn errors of metabolism identified 114 affected newborns, including 55 Emiratis, with the most common conditions being biotinidase deficiency, phenylketonuria, and 3-methylcrotonyl glycinuria. Mutation analysis revealed 33 clinically significant variants, predominantly homozygous, highlighting the burden of autosomal recessive disorders and reinforcing the clinical impact of integrating genomic screening into existing newborn and premarital screening programs in the UAE (70).

Globally, several pilot initiatives demonstrate the feasibility and potential impact of genomic screening. For example, the BabySeq Project in the United States has shown that sequencing healthy and ill newborns can yield medically actionable findings in 9–11% of cases, with strong parental interest and minimal distress when results are appropriately returned with genetic counseling (71, 72, 73). Compared to this model, the UAE benefits from a centralized national healthcare system, a high consanguinity rate, and an established newborn screening infrastructure, which together create a uniquely favorable environment for expanding to universal genomic screening provided ethical and legal frameworks continue to be upheld.

Implementing such a program would have far-reaching implications for clinical practice and health policy. Clinically, it necessitates updated care pathways that integrate genomic information into pediatric and adult medicine, enabling risk-informed decisions from birth. From a public health perspective, the model aligns with a preventive care philosophy and supports national strategies targeting inherited and vaccine preventable diseases. On a systems level, success will depend on building robust digital infrastructure, including secure data platforms and bioinformatics powered tools to support continuous clinical learning and decision making. Clear ethical and legal frameworks must also be established to ensure proper data governance, privacy protection, and equitable access. Effective implementation will require multisector collaboration among geneticists, clinicians, public health experts, and policymakers. Once established, this model can be adapted by countries with similar genetic and demographic profiles to meet their specific health goals.

## Conclusion

This perspective supports universal genomic screening as a practical and necessary strategy to integrate known pathogenic alleles, such as those causing MSMD into routine clinical care, particularly in high-consanguinity populations. Using MSMD and the UAE as a proof of concept, we illustrate how early identification of genetic risk can inform targeted interventions, improve vaccine safety, reduce diagnostic delays, and strengthen preventive healthcare across the lifespan. A sequential genomic screening approach, supported by the UAE’s existing screening infrastructure, national genomic initiatives, and legal frameworks, offers a feasible and cost-effective model for implementation. Expanding this approach into a life-course genomic strategy would not only improve outcomes for MSMD but also serve as a foundation for addressing other IEIs through precision medicine and prevention. Moreover, this model may contribute to the enrichment of underrepresented Arab genomic datasets, addressing a critical gap in global knowledge while facilitating early identification of clinically significant variants in anticipation of future large-scale studies. Looking forward, integrating genomic tools into healthcare systems can enable countries to build resilient and data-driven infrastructures that support equitable, preventive, and personalized medicine across diverse populations.

## Data Availability

No new data were generated or analyzed in support of this study.

## References

[1] Adams, D.R., and C.M. Eng. 2018. Next-Generation sequencing to diagnose suspected genetic disorders. N. Engl. J. Med. 379:1353–1362. 10.1056/NEJMra1711801 30281996

[2] Wright, C.F., P. Campbell, R.Y. Eberhardt, S. Aitken, D. Perrett, S. Brent, P. Danecek, E.J. Gardner, V.K. Chundru, S.J. Lindsay, . 2023. Genomic diagnosis of rare pediatric disease in the United Kingdom and Ireland. N. Engl. J. Med. 388:1559–1571. 10.1056/NEJMoa2209046 37043637 PMC7614484

[3] Aziz Bousfiha, A., L. Jeddane, A. Moundir, M. Cecilia Poli, I. Aksentijevich, C. Cunningham-Rundles, S. Hambleton, C. Klein, T. Morio, C. Picard, . 2025. The 2024 update of IUIS phenotypic classification of human inborn errors of immunity. J. Hum. Immun. 1:e20250002. 10.70962/jhi.20250002 41608113 PMC12829316

[4] Cortesi, M., L. Dotta, M. Cattalini, V. Lougaris, A. Soresina, and R. Badolato. 2024. Unmasking inborn errors of immunity: Identifying the red flags of immune dysregulation. Front. Immunol. 15:1497921. 10.3389/fimmu.2024.1497921 39749336 PMC11693724

[5] Thalhammer, J., G. Kindle, A. Nieters, S. Rusch, M.R.J. Seppänen, A. Fischer, B. Grimbacher, D. Edgar, M. Buckland, N. Mahlaoui, . 2021. Initial presenting manifestations in 16,486 patients with inborn errors of immunity include infections and noninfectious manifestations. J. Allergy Clin. Immunol. 148:1332–1341.e5. 10.1016/j.jaci.2021.04.015 33895260

[6] Tsoulis, M.W., and K.W. Williams. 2025. Keeping up with recent developments in immunodeficiency. Ann. Allergy Asthma Immunol. 134:259–268. 10.1016/j.anai.2024.12.016 39716531

[7] Tadmouri, G.O., P. Nair, T. Obeid, M.T. Al Ali, N. Al Khaja, and H.A. Hamamy. 2009. Consanguinity and reproductive health among Arabs. Reprod. Health. 6:17. 10.1186/1742-4755-6-17 19811666 PMC2765422

[8] Albesher, N., S. Massadeh, S.M. Hassan, and M. Alaamery. 2022. Consanguinity and congenital heart disease susceptibility: Insights into rare genetic variations in Saudi Arabia. Genes. 13:354. 10.3390/genes13020354 35205398 PMC8871910

[9] Elliott, K.S., M. Haber, H. Daggag, G.B. Busby, R. Sarwar, D. Kennet, M. Petraglia, L.J. Petherbridge, P. Yavari, F.U. Heard-Bey, . 2022. Fine-scale genetic structure in the United Arab Emirates reflects ndogamous and consanguineous culture, population history, and geography. Mol. Biol. Evol. 39:msac039. 10.1093/molbev/msac039 35192718 PMC8911814

[10] Federal Competitiveness and Statistics Centre . 2025. Statistics by subject. https://fcsc.gov.ae/en-us/Pages/Statistics/Statistics-by-Subject.aspx (accessed July 17, 2025).

[11] Almarzooqi, F., A.-K. Souid, R. Vijayan, and S. Al-Hammadi. 2021. Novel genetic variants of inborn errors of immunity. PloS One. 16:e0245888. 10.1371/journal.pone.0245888 33481921 PMC7822508

[12] Ministry of Health and Prevention (UAE) . 2024. Genetic testing as part of premarital screening for Emiratis. https://mohap.gov.ae/en/w/genetic-testing-as-part-of-premarital-screening-for-emiratis (accessed July 17, 2025).

[13] UAE Government . 2024. Children’s Health. https://u.ae/en/information-and-services/health-and-fitness/childrens-health (accessed July 17, 2025).

[14] Alswaidi, F.M., and S.J. O’Brien. 2009. Premarital screening programmes for haemoglobinopathies, HIV and hepatitis viruses: Review and factors affecting their success. J. Med. Screen. 16:22–28. 10.1258/jms.2008.008029 19349527

[15] Kalyta, K., W. Stelmaszczyk, D. Szczęśniak, L. Kotuła, P. Dobosz, and M. Mroczek. 2023. The spectrum of the heterozygous effect in biallelic mendelian diseases-the symptomatic heterozygote issue. Genes. 14:1562. 10.3390/genes14081562 37628614 PMC10454578

[16] Zschocke, J., P.H. Byers, and A.O.M. Wilkie. 2023. Mendelian inheritance revisited: Dominance and recessiveness in medical genetics. Nat. Rev. Genet. 24:442–463. 10.1038/s41576-023-00574-0 36806206

[17] Khavandegar, A., S.A. Mahdaviani, M. Zaki-Dizaji, F. Khalili-Moghaddam, S. Ansari, S. Alijani, N. Taherzadeh-Ghahfarrokhi, D. Mansouri, J.-L. Casanova, J. Bustamante, and M. Jamee. 2024. Genetic, immunologic, and clinical features of 830 patients with mendelian susceptibility to mycobacterial diseases (MSMD): A systematic review. J. Allergy Clin. Immunol. 153:1432–1444. 10.1016/j.jaci.2024.01.021 38341181 PMC11880893

[18] Boisson-Dupuis, S., and J. Bustamante. 2021. Mycobacterial diseases in patients with inborn errors of immunity. Curr. Opin. Immunol. 72:262–271. 10.1016/j.coi.2021.07.001 34315005 PMC9172628

[19] Government of the United Arab Emirates . 2024. Children’s health.. https://u.ae/en/information-and-services/health-and-fitness/childrens-health (accessed July 14, 2025).

[20] Al-Herz, W., E.H. Husain, M. Adeli, T. Al Farsi, S. Al-Hammadi, A.A. Al Kuwaiti, M. Al-Nesf, N. Al Sukaiti, S. Al-Tamemi, and H. Shendi. 2022. BCG vaccine-associated complications in a large cohort of children with combined immunodeficiencies affecting cellular and humoral immunity. Pediatr. Infect. Dis. J. 41:933–937. 10.1097/INF.0000000000003678 36102730

[21] Al-Hammadi, S., A.R. Alsuwaidi, E.T. Alshamsi, G.A. Ghatasheh, and A.-K. Souid. 2017. Disseminated Bacillus Calmette-Guérin (BCG) infections in infants with immunodeficiency. BMC Res. Notes. 10:177. 10.1186/s13104-017-2499-7 28476145 PMC5420150

[22] Al-Hammadi, S., N.S. Alkuwaiti, G.A. Ghatasheh, H. Al Dhanhani, H.M. Shendi, A.S. Elomami, F. Almarzooqi, and A.-K. Souid. 2020. Adverse events of the BCG (Bacillus calmette-guérin) and rotavirus vaccines in a young infant with inborn error of immunity. Case Rep. Immunol. 2020:8857152. 10.1155/2020/8857152 PMC7737464 33354374

[23] Al-Hammadi, S., A.M. Yahya, A. Al-Amri, A. Shibli, G.B. Balhaj, M.I. Tawil, R. Vijayan, and A.-K. Souid. 2021. Case report: BCG-triggered hemophagocytic lymphohistiocytosis in an infant with X-linked recessive mendelian susceptibility to mycobacterial disease due to a variant of chronic granulomatous disease. Front. Pediatr. 9:687538. 10.3389/fped.2021.687538 34268280 PMC8275851

[24] Bukhari, E., F. Alaklobi, H. Bakheet, A. Alrabiaah, F. Alotibi, F. Aljobair, M. Alshamrani, E. Alaki, F. Alnahdi, A. Alodyani, and F. Alzamil. 2016. Disseminated bacille calmette-Guérin disease in Saudi children: Clinical profile, microbiology, immunology evaluation and outcome. Eur. Rev. Med. Pharmacol. Sci. 20:3696–3702. 27649674

[25] Prando, C., A. Samarina, J. Bustamante, S. Boisson-Dupuis, A. Cobat, C. Picard, Z. AlSum, S. Al-Jumaah, S. Al-Hajjar, H. Frayha, . 2013. Inherited IL-12p40 deficiency: Genetic, immunologic, and clinical features of 49 patients from 30 kindreds. Medicine. 92:109–122. 10.1097/MD.0b013e31828a01f9 23429356 PMC3822760

[26] Aldhaheri, A., O. Alyabes, S. Aljumaah, R. Alhuthil, R. Alonazi, S. Alamoudi, M. Alsuhaibani, S. Alghamdi, E.A. Albanyan, S. Al-Hajjar, . 2025. The effects of postponing BCG vaccination on the risk of BCG-related complications among patients with severe combined immunodeficiency disease in Saudi Arabia. Front. Immunol. 16:1596963. 10.3389/fimmu.2025.1596963 40416983 PMC12098544

[27] Errami, A., J.E. Baghdadi, F. Ailal, I. Benhsaien, J.E. Bakkouri, L. Jeddane, N. Rada, N. Benajiba, K. Mokhantar, K. Ouazahrou, . 2023. Mendelian susceptibility to mycobacterial disease (MSMD): Clinical, immunological, and genetic features of 22 patients from 15 Moroccan kindreds. J. Clin. Immunol. 43:728–740. 10.1007/s10875-022-01419-x 36630059 PMC10121882

[28] Al Waili, B., N. Al Mufarajii, S. Al Hashmi, A. Al Ajmi, and N. Al Sukaiti. 2021. Bacillus Calmette-Guérin vaccine-related complications in children in Oman. Ann. Saudi Med. 41:24–30. 10.5144/0256-4947.2021.24 33550906 PMC7868619

[29] World Health Organization, Regional Office for the Eastern Mediterranean . 2022. Epidemiological situation of tuberculosis. https://www.emro.who.int/tuberculosis/epidemiological-situation/index.html (accessed July 16, 2025).

[30] Almarzooqi, F., A. Alkhemeiri, A. Aljaberi, R. Hashmey, T. Zoubeidi, and A.-K. Souid. 2018. Prospective cross-sectional study of tuberculosis screening in United Arab Emirates. Int. J. Infect. Dis. 70:81–85. 10.1016/j.ijid.2018.03.001 29526607

[31] Al Mekaini, L.A., O.N. Al Jabri, H. Narchi, S.M. Kamal, A. Mabrook, M.M. Al Kuwaiti, M.M. Sheek-Hussein, A.-K. Souid, and A.R. Alsuwaidi. 2014. The use of an interferon-gamma release assay to screen for pediatric latent tuberculosis infection in the eastern region of the Emirate of Abu Dhabi. Int. J. Infect. Dis. 23:4–7. 10.1016/j.ijid.2013.12.020 24657274

[32] Sheek-Hussein, M., R. Hashmey, A.R. Alsuwaidi, F. Al Maskari, L. Amiri, and A.-K. Souid. 2012. Seroprevalence of measles, mumps, rubella, varicella-zoster and hepatitis A-C in Emirati medical students. BMC Public Health. 12:1047. 10.1186/1471-2458-12-1047 23217121 PMC3526402

[33] Miller, R.D., S. Duan, E.G. Lovins, E.F. Kloss, and P.-Y. Kwok. 2003. Efficient high-throughput resequencing of genomic DNA. Genome Res. 13:717–720. 10.1101/gr.886203 12654721 PMC430165

[34] Wang, L., C. Zhang, J. Watkins, Y. Jin, M. McNutt, and Y. Yin. 2016. SoftPanel: A website for grouping diseases and related disorders for generation of customized panels. BMC Bioinformatics. 17:153. 10.1186/s12859-016-0998-5 27044653 PMC4820874

[35] Lee, I.-H., Y. Lin, W.J. Alvarez, C. Hernandez-Ferrer, K.D. Mandl, and S.W. Kong. 2021. WEScover: Selection between clinical whole exome sequencing and gene panel testing. BMC Bioinformatics. 22:259. 10.1186/s12859-021-04178-5 34016036 PMC8139020

[36] Chen, E., F.M. Facio, K.W. Aradhya, S. Rojahn, K.E. Hatchell, S. Aguilar, K. Ouyang, S. Saitta, A.K. Hanson-Kwan, N.N. Capurro, . 2023. Rates and classification of variants of uncertain significance in hereditary disease genetic testing. JAMA Netw. Open. 6:e2339571. 10.1001/jamanetworkopen.2023.39571 37878314 PMC10600581

[37] Richards, S., N. Aziz, S. Bale, D. Bick, S. Das, J. Gastier-Foster, W.W. Grody, M. Hegde, E. Lyon, E. Spector, . 2015. Standards and guidelines for the interpretation of sequence variants: A joint consensus recommendation of the American College of medical genetics and genomics and the association for molecular pathology. Genet. Med. 17:405–424. 10.1038/gim.2015.30 25741868 PMC4544753

[38] Lek, M., K.J. Karczewski, E.V. Minikel, K.E. Samocha, E. Banks, T. Fennell, A.H. O’Donnell-Luria, J.S. Ware, A.J. Hill, B.B. Cummings, . 2016. Analysis of protein-coding genetic variation in 60,706 humans. Nature. 536:285–291. 10.1038/nature19057 27535533 PMC5018207

[39] Scholtz, D., T. Jooste, M. Möller, A. van Coller, C. Kinnear, and B. Glanzmann. 2023. Challenges of diagnosing mendelian susceptibility to mycobacterial diseases in South Africa. Int. J. Mol. Sci. 24:12119. 10.3390/ijms241512119 37569495 PMC10418440

[40] Vorsteveld, E.E., C.I. Van der Made, S.P. Smeekens, J.H. Schuurs-Hoeijmakers, G. Astuti, H. Diepstra, C. Gilissen, E. Hoenselaar, A. Janssen, K. van Roozendaal, . 2024. Clinical exome sequencing data from patients with inborn errors of immunity: Cohort level diagnostic yield and the benefit of systematic reanalysis. Clin. Immunol. 268:110375. 10.1016/j.clim.2024.110375 39369972

[41] Li, L., X. Tian, V. Woodzell, R.A. Gibbs, B. Yuan, and E. Venner. 2024. Tracking updates in clinical databases increases efficiency for variant reanalysis. Genet. Med. Open. 2:101841. 10.1016/j.gimo.2024.101841 39669589 PMC11613846

[42] Hiatt, S.M., M.D. Amaral, K.M. Bowling, C.R. Finnila, M.L. Thompson, D.E. Gray, J.M.J. Lawlor, J.N. Cochran, E.M. Bebin, K.B. Brothers, . 2018. Systematic reanalysis of genomic data improves quality of variant interpretation. Clin. Genet. 94:174–178. 10.1111/cge.13259 29652076 PMC5995667

[43] Bennett, G., I. Karbassi, W. Chen, S.M. Harrison, M.S. Lebo, L. Meng, N. Nagan, R. Rigobello, and H.L. Rehm. 2025. Distinct rates of VUS reclassification are observed when subclassifying VUS by evidence level. Genet. Med. 27:101400. 10.1016/j.gim.2025.101400 40035215 PMC12181064

[44] Walsh, N., A. Cooper, A. Dockery, and J.J. O’Byrne. 2024. Variant reclassification and clinical implications. J. Med. Genet. 61:207–211. 10.1136/jmg-2023-109488 38296635

[45] Anderson, D., and T. Lassmann. 2022. An expanded phenotype centric benchmark of variant prioritisation tools. Hum. Mutat. 43:539–546. 10.1002/humu.24362 35224813 PMC9313608

[46] Chinn, I.K., A.Y. Chan, K. Chen, J. Chou, M.J. Dorsey, J. Hajjar, A.M. Jongco, K.E. Sullivan, T.K. Tarrant, T.R. Torgerson, . 2020. Diagnostic interpretation of genetic studies in patients with primary immunodeficiency diseases: A working group report of the primary immunodeficiency diseases committee of the American academy of allergy, asthma & immunology. J. Allergy Clin. Immunol. 145:46–69. 10.1016/j.jaci.2019.09.009 31568798

[47] Zouk, H., W. Yu, A. Oza, M. Hawley, P.K. Vijay Kumar, C. Koch, L.M. Mahanta, J.B. Harley, G.P. Jarvik, E.W. Karlson, . 2022. Reanalysis of eMERGE phase III sequence variants in 10,500 participants and infrastructure to support the automated return of knowledge updates. Genet. Med. 24:454–462. 10.1016/j.gim.2021.10.010 34906510 PMC10128874

[48] Clinical Genome Resource (ClinGen) . 2025. Sequence variant interpretation working group. https://clinicalgenome.org/working-groups/sequence-variant-interpretation/(accessed August 24, 2025).

[49] National Cancer Institute . 2023. Genetic Testing Fact Sheet. https://www.cancer.gov/about-cancer/causes-prevention/genetics/genetic-testing-fact-sheet (accessed August 24, 2025).

[50] Hodge, S.E., and D.A. Greenberg. 2016. How can we explain very low odds ratios in GWAS? I. Polygenic models. Hum. Hered. 81:173–180. 10.1159/000454804 28171865

[51] Wald, N.J., and R. Old. 2019. The illusion of polygenic disease risk prediction. Genet. Med. 21:1705–1707. 10.1038/s41436-018-0418-5 30635622

[52] Statista . 2024. Crude birth rate in the United Arab Emirates from 2013 to 2023. https://www.statista.com/statistics/977328/crude-birth-rate-in-the-united-arab-emirates/(accessed July 17, 2025).

[53] Emirati Genome Program . 2023. About the Emirati genome program. https://emiratigenomeprogram.ae/en/index.html (accessed July 17, 2025).

[54] Al Busaidi, N., P. Kp, A. Al-Jardani, N. Al-Sukaiti, S. Al Tamemi, B. Al-Rawahi, Z. Al Hinai, F. Alyaquobi, S. Al-Abri, and A. Al-Maani. 2021. The spectrum of bacille Calmette-Guérin diseases in children-A decade of data from neonatal vaccination settings. Vaccines. 9:150. 10.3390/vaccines9020150 33668524 PMC7917994

[55] Al-Hajoj, S., Z. Memish, N. Abuljadayel, R. AlHakeem, F. AlRabiah, and B. Varghese. 2014. Molecular confirmation of Bacillus Calmette Guerin vaccine related adverse events among Saudi Arabian children. PloS One. 9:e113472. 10.1371/journal.pone.0113472 25409184 PMC4237431

[56] Ghaleb, W., Y. Aljishi, S. Ayathan, and A. Almemari. 2023. Cost evaluation of current pulmonary MTB diagnosis process in a hospital in Abu Dhabi and proposal to implement the World health Organization MTB clinical pathway. Eurasian J. Emerg. Med. 22:93–98. 10.4274/eajem.galenos.2023.48992

[57] Stadtmauer, G., and C. Cunningham-Rundles. 1997. Outcome analysis and cost assessment in immunologic disorders. JAMA. 278:2018–2023. 9396665

[58] DNA Labs UAE . 2024. Whole exome sequencing (WES) test. https://dnalabsuae.com/tests/whole-exome-sequencing-wes-test/(accessed July 17, 2025).

[59] King, J.R., L.D. Notarangelo, and L. Hammarström. 2021. An appraisal of the Wilson & Jungner criteria in the context of genomic-based newborn screening for inborn errors of immunity. J. Allergy Clin. Immunol. 147:428–438. 10.1016/j.jaci.2020.12.633 33551024 PMC8344044

[60] Blom, M., R.G.M. Bredius, and M. van der Burg. 2023. Efficient screening strategies for severe combined immunodeficiencies in newborns. Expert Rev. Mol. Diagn. 23:815–825. 10.1080/14737159.2023.2244879 37599592

[61] Yang, Y., L. Xia, and S. Lu. 2023. Adult-onset mendelian susceptibility to mycobacterial diseases: A case report and systematic literature review. Heliyon. 9:e22632. 10.1016/j.heliyon.2023.e22632 38058431 PMC10696185

[62] Alcaïs, A., C. Fieschi, L. Abel, and J.-L. Casanova. 2005. Tuberculosis in children and adults: Two distinct genetic diseases. J. Exp. Med. 202:1617–1621. 10.1084/jem.20052302 16365144 PMC2212964

[63] Stark, Z., and R.H. Scott. 2023. Genomic newborn screening for rare diseases. Nat. Rev. Genet. 24:755–766. 10.1038/s41576-023-00621-w 37386126

[64] Jiang, S., H. Wang, and Y. Gu. 2023. Genome sequencing for newborn screening-an effective approach for tackling rare diseases. JAMA Netw. Open. 6:e2331141. 10.1001/jamanetworkopen.2023.31141 37656463

[65] Baple, E.L., R.H. Scott, S. Banka, J. Buchanan, L. Fish, S. Wynn, D. Wilkinson, S. Ellard, D.G. MacArthur, and Z. Stark. 2024. Exploring the benefits, harms and costs of genomic newborn screening for rare diseases. Nat. Med. 30:1823–1825. 10.1038/s41591-024-03055-x 38898121

[66] United Arab Emirates Government . 2016. Federal Law No. 4 of 2016 on Medical Liability. https://uaelegislation.gov.ae/en/legislations/1192/(accessed July 16, 2025).

[67] United Arab Emirates Government . 2021. Health Data Law (Use of Information and Communications Technology in Healthcare). https://uaelegislation.gov.ae/en/legislations/1209/(accessed July 16, 2025).

[68] United Arab Emirates Government . 2023. Federal Decree-Law No. 2195 Regulating the Use of the Human Genome. https://uaelegislation.gov.ae/en/legislations/2195/(accessed July 16, 2025).

[69] Elhadi, Y.A.M., M. Alkatheeri, M. Alktifan, F. Alhammadi, T. Sultan, Y.M.A. Alqumboz, A. Jihad, M.I. Shaidul, M. Al Saadi, M.S.N. Alkaabi, . 2025. Parents' perspectives on expanded newborn genomic screening in Abu Dhabi, United Arab Emirates. Hum. Genomics. 19:63. 10.1186/s40246-025-00766-1 40481610 PMC12144800

[70] Al-Jasmi, F.A., A. Al-Shamsi, J.L. Hertecant, S.M. Al-Hamad, and A.-K. Souid. 2016. Inborn errors of metabolism in the United Arab Emirates: Disorders detected by newborn screening (2011–2014). JIMD Rep. 28:127–135. 10.1007/8904_2015_512 26589311 PMC5059198

[71] Holm, I.A., P.B. Agrawal, O. Ceyhan-Birsoy, K.D. Christensen, S. Fayer, L.A. Frankel, C.A. Genetti, J.B. Krier, R.C. LaMay, H.L. Levy, . 2018. The BabySeq project: Implementing genomic sequencing in newborns. BMC Pediatr. 18:225. 10.1186/s12887-018-1200-1 29986673 PMC6038274

[72] Green, R.C., N. Shah, C.A. Genetti, T. Yu, B. Zettler, M.K. Uveges, O. Ceyhan-Birsoy, M.S. Lebo, S. Pereira, P.B. Agrawal, . 2023. Actionability of unanticipated monogenic disease risks in newborn genomic screening: Findings from the BabySeq Project. Am. J. Hum. Genet. 110:1034–1045. 10.1016/j.ajhg.2023.05.007 37279760 PMC10357495

[73] Smith, H.S., B. Zettler, C.A. Genetti, M.R. Hickingbotham, T.F. Coleman, M. Lebo, A. Nagy, H. Zouk, L. Mahanta, K.D. Christensen, . 2024. The BabySeq project: A clinical trial of genome sequencing in a diverse cohort of infants. Am. J. Hum. Genet. 111:2094–2106. 10.1016/j.ajhg.2024.08.011 39288765 PMC11480845

